# Inequality in quality-adjusted life expectancy by educational attainment in Norway: an observational study

**DOI:** 10.1186/s12889-023-15663-2

**Published:** 2023-05-03

**Authors:** Nils Gutacker, Jonas Minet Kinge, Jan Abel Olsen

**Affiliations:** 1 Centre for Health Economics, University of York, Alcuin A Block, Heslington, YO10 5DD UK; 2 Norwegian Institute of Public Health, Oslo, Norway; 3 Department of Health Management and Health Economics, University of Oslo, Oslo, Norway; 4 Department of Community Medicine, UiT - the Arctic University of Norway, Tromsø, Norway

**Keywords:** Health inequality, Health-related quality of life, Educational attainment, Quality adjusted life expectancy, Norway

## Abstract

**Background:**

Health inequalities are often assessed in terms of life expectancy or health-related quality of life (HRQoL). Few studies combine both aspects into quality-adjusted life expectancy (QALE) to derive comprehensive estimates of lifetime health inequality. Furthermore, little is known about the sensitivity of estimated inequalities in QALE to different sources of HRQoL information. This study assesses inequalities in QALE by educational attainment in Norway using two different measures of HRQoL.

**Methods:**

We combine full population life tables from Statistics Norway with survey data from the Tromsø study, a representative sample of the Norwegian population aged ≥ 40. HRQoL is measured using the EQ-5D-5L and EQ-VAS instruments. Life expectancy and QALE at 40 years of age are calculated using the Sullivan-Chiang method and are stratified by educational attainment. Inequality is measured as the absolute and relative gap between individuals with lowest (i.e. primary school) and highest (university degree 4 + years) educational attainment.

**Results:**

People with the highest educational attainment can expect to live longer lives (men: + 17.9% (95%CI: 16.4 to 19.5%), women: + 13.0% (95%CI: 10.6 to 15.5%)) and have higher QALE (men: + 22.4% (95%CI: 20.4 to 24.4%), women: + 18.3% (95%CI: 15.2 to 21.6%); measured using EQ-5D-5L) than individuals with primary school education. Relative inequality is larger when HRQoL is measured using EQ-VAS.

**Conclusion:**

Health inequalities by educational attainment become wider when measured in QALE rather than LE, and the degree of this widening is larger when measuring HRQoL by EQ-VAS than by EQ-5D-5L. We find a sizable educational gradient in lifetime health in Norway, one of the most developed and egalitarian societies in the world. Our estimates provide a benchmark against which other countries can be compared.

**Supplementary Information:**

The online version contains supplementary material available at 10.1186/s12889-023-15663-2.

## Key points

- Differences in quality-adjusted life expectancy (QALE) among population groups are under-studied.
- Higher educational attainment is associated with higher life expectancy and better health-related quality of life in Norway.
- University-educated Norwegian have approximately 18–27% higher QALE than those with only primary or lower secondary school education.
- QALE estimates are sensitive to the choice of HRQoL instrument.

## Background

Systematic inequalities in lifetime health across individuals of different socioeconomic backgrounds have been documented in many countries [1] and are widely perceived as unfair [2]. Any attempt to quantify inequalities requires measurement; and while the exact definition of lifetime health remains somewhat elusive, most commonly used summary measures acknowledge that it involves a combination of quantity (i.e. how long someone lives) and quality (how well they live) [3]. It follows that the measured degree of socioeconomic inequality in lifetime health in a society depends on the health indicator used and how well it captures both these elements.

Most studies of socioeconomic inequalities in health have focus on life expectancy (LE), i.e., the *quantity* aspect, which can often be calculated from full population birth and death registries further disaggregated by educational attainment [4], income [5] or wealth [6]. Another strand of the literature quantifies socioeconomic inequalities in health-related *quality* of life (HRQoL), either directly collected through validated questionnaires as part of population health surveys [7, 8] or indirectly approximated through markers of morbidity. However, only few studies seek to quantify socioeconomic inequalities in lifetime health by combining elements of LE and HRQoL into a comprehensive summary measure such as quality-adjusted life expectancy (QALE) [9–13]. QALE is the sum of quality-adjusted life years (QALYs) an individual can expect over their remaining lifetime where each year lived is weighted by the expected HRQoL enjoyed during this year. If inequalities in LE and HRQoL operate in the same direction (e.g. favour those of higher socioeconomic position), partial estimates of socioeconomic inequalities based on either LE *or* HRQoL are likely to underestimate joint inequalities. Sex difference may be particularly relevant in this context: While women typically have higher average LE than men, socioeconomic inequalities are often lower [14]. Women also tend to report lower HRQoL than men [15] although this may not necessarily translate into different socioeconomic inequalities [16].

The aims of this study are twofold: First, we seek to quantify inequalities in LE and QALE by educational attainment in Norway and how these differ across sexes, thereby adding to a small literature on inequalities in QALE by educational attainment [10, 13]. Norway is a high-income nation with generous social security system, and with more evenly distributed income and wealth than most OECD countries. It has a tax-funded national health service with minimal co-payments, and equal opportunities to enter publicly financed tertiary education, thereby limiting the influence of two known determinants of inequality [17]. Hence, Norway offers a useful ‘best-case’ benchmark against which other countries can be compared. However, inequalities in QALE have not been quantified for Norway, yet.

Second, we investigate how sensitive estimates of QALE inequality are to the source of HRQoL weights used. Several different summary measures of lifetime health have been proposed and compared, including QALE, disability-adjusted life expectancy, and healthy life expectancy [3]. In contrast, relatively little is known about the effect of using different sources of HRQoL data to compute QALE, with only one previous study exploring this issue [18]. In this study, we compare QALE inequality estimates calculated using quality weights obtained from the EQ-5D-5L instrument, a multi-attribute utility measure widely used in health technology assessment that provides indirect valuations of respondents’ HRQoL based on a health state description, and the EQ-VAS instrument, a visual analogue scale that provides direct valuations of respondents’ HRQoL.

## Methods

### Data sources

We combined information from two sources.

#### HRQoL data

HRQoL data was obtained from The Tromsø Study which is a long-running, prospective cohort study of the general population residing in the largest city of Northern Norway. The cohort is considered to be broadly representative of the general adult population of Norway, although it is skewed towards more educated university graduates. Further details on the study design have been reported elsewhere [19].

The 7th wave of the Tromsø Study (2015/16) asked all residents aged 40 and older to report their HRQoL using the EQ-5D-5L instrument, which is a generic preference-based measure suitable for population health assessment [20]. Participants described their HRQoL along five dimensions: mobility, self-care, usual activities, pain & discomfort, and anxiety & depression. For each dimension, participants could indicate whether they experience no, slight, moderate, severe, or extreme problems. Together these responses form a participant’s EQ-5D-5L health profile. In lieu of an official scoring algorithm for Norway, we used a recently published amalgam estimate of preferences over health profiles based on the value sets from ten Western countries (Canada, Denmark, England, France, Germany, Republic of Ireland, the Netherlands, Portugal, Spain, US) (the ‘Western Preference Pattern’ (MN-WePP) value set [21]. The resulting index scores range from 1 (full health) to -0.578, where scores below zero indicate health profiles considered worse than being dead. Participants were also asked to report their HRQoL on a visual analogue scale (the EQ-VAS), with endpoints being defined as worst (scored as zero) and best imaginable health (scored as 100; rescaled to 1).

Age at the time of survey was coded in 5-year age bands, with participants aged 75 or over grouped together due to low numbers. Participants’ highest educational attainment was categorised in line with the International Standard Classification of Education (ISCED): primary and lower secondary (L1); vocational and upper secondary (L2); college and university - less than 4 years (L3); and college and university - 4 years or more (L4).

#### Population and death data

Counts of population and deaths by age, sex and educational attainment level for the period 01 January to 31 December 2016 were obtained from linked individual-level information from the Population Register [22], the Cause of Death Registry [23], and the National Education Database [24]. The linked dataset contains complete information on education and population for more than 99% of the official Norwegian population above the age of 40.

### Statistical analysis

QALE at age 40 was estimated by combining mortality rates with HRQoL information. Mortality rates in each age-sex-education group up to the age of 95 were derived from population statistics. For ages 96 to 106 we used sex-specific mortality rates published by Statistics Norway due to small cell sizes by educational attainment. The Chiang II method [25] was used to calculate period life tables. All individuals were assumed to die at the end of their 106th year, implying a maximum modelled LE of 66 years. Average HRQoL by 5-year age group (coded as 40–44, 45–49, 50–54, 55–59, 60–64, 65–69, 70–74, 75 and over), sex and educational attainment was calculated and used to adjust LE for differences in HRQoL using the Sullivan method [26]. The resulting QALE estimates reflects the number of QALYs that a person aged 40 of a given sex and educational attainment can expect to experience over their remaining lifetime up the age of 106. See Online Supplementary Material for a detailed description of the calculations conducted.

Inequalities in LE and QALE were measured as absolute and relative gaps between individuals with the highest (L4) and lowest (L1) educational attainment. The absolute gap is simply the difference between the LE or QALE estimates between the two groups. The relative gap is the ratio of LE or QALE in L4 to L1 and measure the percentage increase in the metric of interest in the highest educational attainment group relative to the level experienced by the lowest educational attainment group.

Uncertainty in QALE was approximated through bootstrapping (1,000 iterations with replacement) of the HRQoL data. In each iteration, mortality risks for each age-sex-education group were resampled from a Beta distribution and life tables were recalculated.

All calculations were performed in Stata 17.

## Results

A total of 21,083 participants took part in wave 7 of the Tromsø Study. Educational attainment could not be ascertained for 378 (1.8%) individuals. Furthermore, EQ-5D-5L profile and EQ-VAS data were missing for 765 (3.6%) and 385 (1.8%) individuals, respectively. This left 19,940 and 20,320 participants for the calculation of mean EQ-5D-5L utilities and VAS scores by age group, sex and educational attainment. The characteristics of this sample are described in Table 1.

**Table 1:** Descriptive statistics of the sample population - Tromsø Study (wave 7, 2015–2016)

	N	%	EQ-5D-5L utility (*N* = 19,940)		EQ-VAS score (*N* = 20,320)	
			Mean	SD	Mean	SD
*Age group*						
40–44	3130	15.0%	0.908	0.109	0.764	0.154
45–49	3264	15.7%	0.903	0.113	0.769	0.157
50–54	3114	15.0%	0.902	0.114	0.772	0.161
55–59	2961	14.2%	0.900	0.115	0.764	0.164
60–64	2704	13.0%	0.906	0.107	0.764	0.160
65–69	2399	11.5%	0.914	0.101	0.772	0.161
70–74	1657	8.0%	0.908	0.114	0.748	0.169
75 +	1576	7.6%	0.884	0.138	0.707	0.179
*Sex*						
Female	10874	52.5%	0.893	0.119	0.759	0.169
Male	9831	47.5%	0.916	0.104	0.764	0.154
*Educational attainment*						
L1 - primary and lower secondary	4796	23.2%	0.887	0.128	0.723	0.179
L2 - vocational and upper secondary	5756	27.8%	0.899	0.113	0.753	0.163
L3 - college and university, < 4 years	4008	19.4%	0.908	0.108	0.769	0.156
L4 - college and university, > = 4 years	6145	29.7%	0.919	0.102	0.794	0.144

**Fig. 1:**
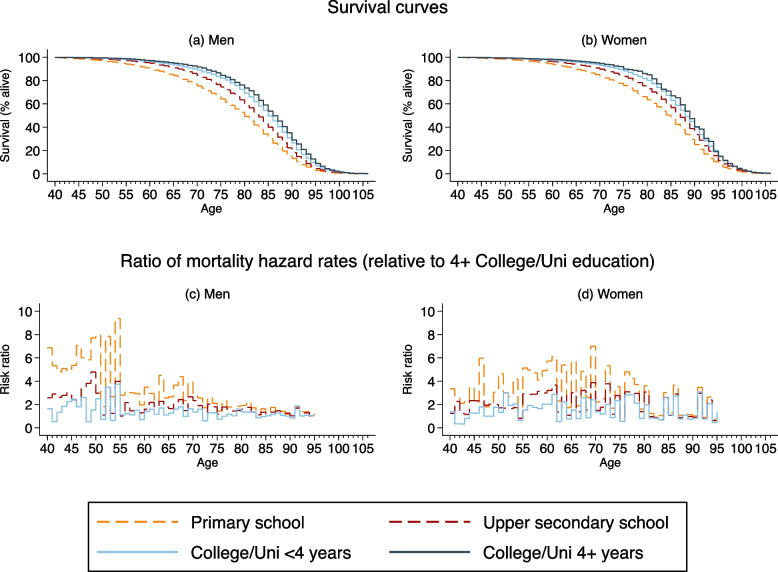
Mortality patterns for men and women, by educational attainment

Figure 1 (panels 1 and 2) demonstrate a clear survival benefit of higher educational attainment. The upper row of Fig. 1 shows survival curves by educational attainment, stratified by sex. Individuals with higher educational attainment enjoy a clear survival benefit over those with lower educational attainment. The lower row of Fig. 1 plot ratios of mortality rates in each educational attainment group to the mortality rates for the 4+ years college and university (L4) group; separately for each sex and up to age 95 before common mortality patterns across educational attainment are assumed. Values > 1 indicate an increased risk of dying at a given year of age compared to similarly aged individuals with L4 educational attainment. There are striking differences between the sexes in these mortality hazard ratios: Men with primary or lower secondary school education (L1) are 6 to 8 times more likely to die in their 40s and 50s than men with the highest education level (L4), and this gap narrows with age. In contrast, the mortality risk ratio increases with age for women up to approximately age 65, when it begins to narrow again.

**Fig. 2:**
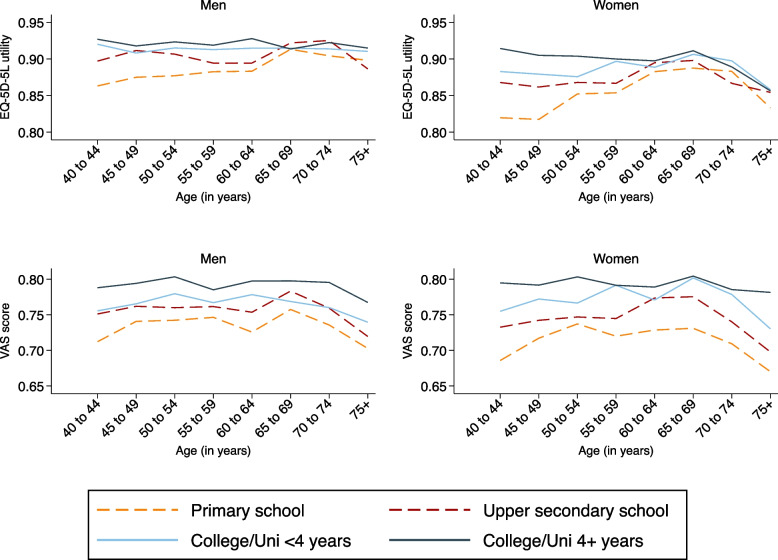
Mean HRQoL scores (EQ-5D-5L, EQ-VAS) by educational attainment, age and sex

Figure 2 show EQ-5D-5L utilities and EQ-VAS scores by 5-year age group, sex and educational attainment. There is a considerable gap in utilities between those with and without a university degree up until approximately age 60 in men and 55 in women when lines begin to converge for both sexes. Furthermore, utilities are markedly lower for women than for men (see also Table 1), and remarkably lower among women aged 40–49 with primary or lower secondary school only (L1). The EQ-VAS data confirm the HRQoL advantage of tertiary educated individuals but show no clear differences in HRQoL by sex.

Table 2 summarises estimates of LE, QALE and their difference (LE minus QALE) at age 40 for the different educational attainment groups, sexes and the two HRQoL measures. Women aged 40 with the highest level of education (L4) can expect to live a further 47.5 years, or approximately 5.5 years (or 13%) more than women of the same age with basic primary or lower secondary school education (L1; 42.1 years). The relative inequalities in LE are even larger for men aged 40 (difference: 6.8 years; or 17.9%). Note the diminishing marginal increases in LE for each additional step on the educational ladder: For men, life expectancy increases by 3.2 years between L1 and L2; by 2.3 years between L2 and L3, and; by 1.3 years between L3 and L4. Similar marginal effects are observed for women.

**Table 2:** Remaining LE and QALE at age 40, by educational attainment and sex

Educational attainment	Men		Women		Men		Women	
	Estimate	95% CI	Estimate	95% CI	Estimate	95% CI	Estimate	95% CI
*Life expectancy (LE) at 40*								
L1 - primary and lower secondary	38.0	(37.7 to 38.2)	42.1	(41.8 to 42.3)				
L2 - vocational and upper secondary	41.2	(41.0 to 41.4)	44.9	(44.7 to 45.1)				
L3 - college and university, < 4 years	43.5	(43.2 to 43.9)	46.6	(46.3 to 47.0)				
L4 - college and university, > = 4 years	44.8	(44.3 to 45.2)	47.5	(46.6 to 48.5)				
Absolute inequality: dLE = L4-L1	6.8	(6.2 to 7.4)	5.5	(4.5 to 6.5)				
Relative inequality: ((L4/L1)-1)*100%	17.9%	(16.4% to 19.5%)	13.0%	(10.6% to 15.5%)				
	HRQoL weight based on EQ-5D-5L utilities				HRQoL weight based on EQ-VAS scores			
*Quality-adjusted life expectancy (QALE)*								
L1 - primary and lower secondary	33.6	(33.3 to 34.0)	35.8	(35.4 to 36.1)	27.7	(27.4 to 28.1)	29.8	(29.4 to 30.2)
L2 - vocational and upper secondary	37.2	(36.9 to 37.4)	39.0	(38.6 to 39.4)	31.0	(30.7 to 31.3)	33.1	(32.7 to 33.5)
L3 - college and university, < 4 years	39.8	(39.3 to 40.2)	41.1	(40.4 to 41.6)	33.1	(32.7 to 33.6)	35.6	(35.0 to 36.3)
L4 - college and university, > = 4 years	41.2	(40.7 to 41.7)	42.3	(41.3 to 43.3)	35.3	(34.7 to 35.8)	37.6	(36.6 to 38.5)
Absolute inequality: dQALE = L4-L1	7.5	(6.9 to 8.1)	6.5	(5.4 to 7.7)	7.5	(6.9 to 8.2)	7.8	(6.7 to 8.8)
Relative inequality: ((L4/L1)-1)*100%	22.4%	(20.4% to 24.4%)	18.3%	(15.2% to 21.6%)	27.1%	(24.6% to 29.9%)	26.3%	(22.4% to 29.9%)
*Quality-adjusted life years (QALYs) lost*								
L1 - primary and lower secondary	4.3	(4.0 to 4.7)	6.3	(5.9 to 6.7)	10.2	(9.8 to 10.6)	12.3	(11.9 to 12.7)
L2 - vocational and upper secondary	4.0	(3.8 to 4.3)	5.9	(5.5 to 6.2)	10.2	(9.9 to 10.5)	11.8	(11.4 to 12.2)
L3 - college and university, < 4 years	3.8	(3.4 to 4.2)	5.6	(5.0 to 6.2)	10.4	(10.0 to 10.9)	11.0	(10.3 to 11.6)
L4 - college and university, > = 4 years	3.6	(3.1 to 4.1)	5.2	(4.2 to 6.3)	9.5	(9.0 to 10.1)	9.9	(9.0 to 10.9)
Absolute inequality: L4-L1	-0.7	(-1.3 to -0.1)	-1.1	(-2.2 to 0.0)	-0.7	(-1.4 to -0.1)	-2.4	(-3.4 to -1.3)
Relative inequality: ((L4/L1)-1)*100%	-16.8%	(-30.0% to -2.5%)	-17.0%	(-34.5% to 0.4%)	-7.1%	(-13.5% to -0.6%)	-19.2%	(-27.1% to -10.3%)

When lifetime health is measured in QALE, the general pattern is that inequalities by educational attainment become wider, and the degree of this widening is larger when measuring HRQoL by EQ-VAS than by EQ-5D-5L. Men in the highest educational attainment level have 7.5 (22%) more remaining QALYs than those in the lowest educational group when measured by EQ-5D-5L, and 7.5 (27%) when measured by EQ-VAS. The corresponding inequalities for women are 6.5 (18%) and 7.8 (26%) QALYs.

It follows that the QALY-equivalent loss due to lower HRQoL (i.e. the difference between LE and QALE) for women is larger when HRQoL is measured by EQ-VAS than by EQ-5D-5L. The absolute inequality in (LE – QALE) between the highest and the lowest education level is 1.1 QALYs (17%) when measured by EQ-5D-5L and 2.4 (19%) when measured by the EQ-VAS. For men, the corresponding absolute inequalities are both 0.7 QALYs but the relative effect is larger when measured using the EQ-VAS (19%) compared to the EQ-5D-5L (17%).

## Discussion

### Main finding

Our study has identified inequalities in lifetime health across educational groups in Norway. People with higher educational attainment will, on average, live longer lives *and* have better HRQoL. Thus, the combined inequalities in QALE are larger than the inequalities in each of the two components of quantity of life (LE) and quality of life (HRQoL) alone. These findings are in line with a small number of previous studies investigating inequalities in QALE by educational level in the Netherlands [10] and South Korea [13].

We observed consistent increases in health inequality by using the QALE metric rather than LE. When comparing LE in the highest level of educational attainment (L4) compared to the lowest level (L1), it was 17.9% higher among men and 13.0% among women. When including HRQoL in the QALE metric, these relative inequalities increased to 22.4 or 27.1% among men, depending on which HRQoL instrument was used (EQ-5D-5L or EQ-VAS), and to 18.3 and 26.3% respectively among women. This is consistent with our finding of HRQoL inequalities favouring higher educated individuals. As a result, adjusting LE for HRQoL amplifies the inequality by educational attainment.

The two associations point in the same direction: the higher the educational level, the longer the LE *and* the better the HRQoL which suggests that longer lives are characterised by a shorter period in ill health with less total lifetime disability [27]. Prior literature suggests various explanations of how education might affect health. First, education might have positive effects on health-related decision-making, thereby increasing individuals’ ability and willingness to control their diet, engage in physical activity, etc. [28, 29]. Second, persons with low education might experience more chronic stress, due to e.g. lower income and or social subordination [30, 31]. Third, education might improve one’s ability to navigate the healthcare system and obtain access to optimal care [32]. Finally, family background, genetic endowments and health shocks during the early stages of life might affect education, e.g. children with poor health often have lower education, which may then result in reduced health later in life [33].

QALE is an appealing metric compared to other population health measures such as disability-free life expectancy because it weighs each life year by a continuous measure of the impact of health disabilities on peoples’ HRQoL [3, 9]. However, HRQoL weights can be derived using different instruments. Our results suggest that QALE estimates are sensitive to this choice. For women, the magnitude of the QALY losses associated with the HRQoL weighting (i.e. LE minus QALE) were generally larger when HRQoL were measured by EQ-VAS as compared to EQ-5D-5L. For men, the opposite was true.

There are several reasons why different instruments may generate different estimates of HRQoL, including scale length, coverage of HRQoL domains, and how instruments are scored [34, 35]. However, one might expect these effects to have similar effects on the estimated inequalities in QALE across males and females and we had therefore not expected this finding. The estimated differential effect of the choice of HRQoL instrument on QALE inequalities by educational attainment cannot be explained with the limited data at hand, and we therefore call for further qualitative and quantitative research to explore this finding.

Multi-attribute utility instruments such as the EQ-5D-5L are often preferred over non-utility-based instrument such as the EQ-VAS when calculating QALYs for the purpose of health technology assessment because of normative properties that permit interpersonal comparisons to inform decisions about which technologies should be funded from a limited healthcare budget. However, these properties are not required when calculating QALYs and QALE for the purpose of population health monitoring. It follows that researchers need to select HRQoL instruments carefully when studying inequalities in QALE.

### Strengths and limitations

The main strength of our analysis is the combination of the large sample size of the Tromsø study, and the availability of full population mortality data that can be broken down by educational attainment up to age 95. Furthermore, by using two HRQoL-measures we show that the magnitude of health inequalities expressed by the QALE-metric crucially depends on how HRQoL is being measured. There are also limitations to our analysis. First, our estimates are based on mortality and HRQoL patterns observed in the Norwegian population in 2016. These are likely to change over time and across cohorts and, consequently, our estimates should be interpreted as period LE and QALE. Second, and closely related, there are strong birth cohort effects in educational attainment, reflecting a strong educational expansion in Norway that took place over the last 50 years and facilitated access to higher education for more recent generations [36]. These cohort effects are particularly pronounced among women, where 44% of individuals aged 70+ in 2016 attended secondary school or higher compared to more than 92% of those aged 40–49. Third, we only observe educational attainment at time of death. Individuals may pursue further education at later stages in their lives, which may bias our estimates of LE if education has a causal effect on mortality. Fourth, we only observed the HRQoL of few individuals aged 75 or over and therefore pooled their estimates. This may lead to an over-estimate of QALE if HRQoL continues to decline as people age. Fifth, our results are likely to be subject to differential survivorship bias by educational attainment, which in turn affects observed HRQoL of the Tromso study population. Specifically, we observe higher mortality rates in individuals with lower educational attainment across nearly all age groups. As a result, HRQoL is measured in an increasingly selected population of survivors with, probably, better health. This might explain why we observe a convergence of HRQoL in EQ-5D utility scores and, to a lesser extent, in EQ-VAS scores across educational attainment in the older age groups. Sixth, our estimates of HRQoL by age are considerably higher than those reported by another recent survey of the general population in Norway [37]. We show in Table S1 in the online supplementary material that this difference to a large extent can be explained by the choice of EQ-5D-5L scoring algorithm, with our study using an international amalgamation of value sets whereas theirs relies on the English value set [38]. There is currently no official EQ-5D-5L value set available for Norway although this is in progress. Seventh, HRQoL data are self-reported and people with different levels of education may report their HRQoL differently [39]. Future research of socio-economic inequalities in QALE may want to adopt methods such as anchoring vignettes to adjust for response heterogeneity [40]. Finally, while the Tromsø study is broadly representative of the Norwegian population in terms of age and sex, it is not geographically representative. However, there are currently no other suitable large-scale HRQoL data collections in Norway that could have been used instead.

## Conclusions

In conclusion, we find a sizable educational gradient in lifetime health in Norway, one of the most developed and egalitarian societies in the world. Our estimates provide a benchmark against which other countries can be compared. Our results emphasise the need to take a holistic approach to measuring health when assessing health inequalities [9]. Researchers need to be aware that estimates of QALE can be sensitive to the source of HRQoL information.

## Supplementary Information

**Additional file 1:** Methods to calculate life tables and QALE.

**Additional file 2: Table S1.** Comparison of EQ-5D-5L utility scores in two surveys of the Norwegian general population calculated using different value sets.

## Data Availability

The data that support the findings of this study can be made available upon reasonable request from Statistics Norway and the Tromsø study team. Restrictions apply to the availability of these data, which were used under license for the current study, and so are not publicly available. The licencing arrangement does not permit the study team to make the data available to others.

## References

[1] Mackenbach JP, Valverde JR, Artnik B, Bopp M, Brønnum-Hansen H, Deboosere P (2018). Trends in health inequalities in 27 European countries. Proc Natl Acad Sci U S A.

[2] McNamara S, Holmes J, Stevely AK, Tsuchiya A (2020). How averse are the UK general public to inequalities in health between socioeconomic groups? A systematic review. Eur J Health Econ.

[3] Hyder AA, Puvanachandra P, Morrow RH (2012). Measuring the health of populations: explaining composite indicators. J Public Health Res.

[4] Kinge JM, Steingrímsdóttir ÓA, Moe JO, Skirbekk V, Næss Ø, Strand BH (2015). Educational differences in life expectancy over five decades among the oldest old in Norway. Age Ageing.

[5] Kinge JM, Modalsli JH, Øverland S, Gjessing HK, Tollånes MC, Knudsen AK (2019). Association of household income with life expectancy and cause-specific mortality in Norway, 2005–2015. JAMA.

[6] Asaria M, Mazumdar S, Chowdhury S, Mazumdar P, Mukhopadhyay A, Gupta I (2019). Socioeconomic inequality in life expectancy in India. BMJ Glob Health.

[7] Teni FS, Gerdtham UG, Leidl R, Henriksson M, Åström M, Sun S (2022). Inequality and heterogeneity in health-related quality of life: findings based on a large sample of cross-sectional EQ-5D-5L data from the Swedish general population. Qual Life Res.

[8] Short H, Al Sayah F, Ohinmaa A, Lahtinen M, Johnson JA (2018). The relationship of neighbourhood-level material and social deprivation with health-related quality of life. Qual Life Res.

[9] Love-Koh J, Asaria M, Cookson R, Griffin S (2015). The social distribution of health: estimating quality-adjusted life expectancy in England. Value Health.

[10] Gheorghe M, Wubulihasimu P, Peters F, Nusselder W, Van Baal PH (2016). Health inequalities in the Netherlands: trends in quality-adjusted life expectancy (QALE) by educational level. Eur J Public Health.

[11] Lim D, Bahk J, Ock M, Kim I, Kang H-Y, Kim Y-Y (2020). Income-related inequality in quality-adjusted life expectancy in Korea at the national and district levels. Health Qual Life Outcomes.

[12] Zhang T, Shi W, Huang Z, Gao D, Guo Z, Chongsuvivatwong V (2016). Gender and ethnic health disparities among the elderly in rural Guangxi, China: estimating quality-adjusted life expectancy. Glob Health Action.

[13] Jo M-W, Seo W, Lim SY, Ock M (2019). The trends in health life expectancy in Korea according to age, gender, education level, and subregion: using quality-adjusted life expectancy method. J Korean Med Sci..

[14] Mackenbach JP, Valverde JR, Bopp M, Brønnum-Hansen H, Deboosere P, Kalediene R (2019). Determinants of inequalities in life expectancy: an international comparative study of eight risk factors. Lancet Public Health.

[15] Jensen MB, Jensen CE, Gudex C, Pedersen KM, Sørensen SS, Ehlers LH (2023). Danish population health measured by the EQ-5D-5L. Scand J Public Health.

[16] Olsen JA, Lindberg MH, Lamu AN (2020). Health and wellbeing in Norway: population norms and the social gradient. Soc Sci Med.

[17] OECD/European Observatory on Health Systems and Policies (2021). Norway: country health profile 2021.

[18] Heijink R, van Baal P, Oppe M, Koolman X, Westert G (2011). Decomposing cross-country differences in quality adjusted life expectancy: the impact of value sets. Popul Health Metr.

[19] Jacobsen BK, Eggen AE, Mathiesen EB, Wilsgaard T, Njølstad I (2012). Cohort profile: the Tromso study. Int J Epidemiol.

[20] Herdman M, Gudex C, Lloyd A, Janssen M, Kind P, Parkin D (2011). Development and preliminary testing of the new five-level version of EQ-5D (EQ-5D-5L). Qual Life Res.

[21] Roudijk B, Janssen B, Olsen JA, Devlin N, Roudijk B, Ludwig K (2022). How do EQ-5D-5L value sets differ?. Value sets for EQ-5D-5L: a compendium, comparative review & user guide.

[22] Norwegian Tax Administration. National population register. Available from: https://www.skatteetaten.no/en/person/national-registry/. Accessed 20 Apr 2023.

[23] Norwegian Institute of Public Health. Norwegian cause of death registry. Available from: https://www.fhi.no/en/hn/health-registries/cause-of-death-registry/. Accessed 20 Apr 2023.

[24] Statistics Norway. About the National Education Database (NUDB). Available from: https://www.ssb.no/a/english/mikrodata/datasamling/nudb/nudb_20130607-en.html. Accessed 20 Apr 2023.

[25] Chiang CL (1972). On constructing current life tables. J Am Stat Assoc.

[26] Sullivan DF (1971). A single index of mortality and morbidity. HSMHA Health Rep.

[27] Fries JF, Bruce B, Chakravarty E (2011). Compression of morbidity 1980–2011: a focused review of paradigms and progress. J Aging Res.

[28] Sobal J (1991). Obesity and socioeconomic status: a framework for examining relationships between physical and social variables. Med Anthropol.

[29] Cutler DM, Lleras-Muney A (2010). Understanding differences in health behaviors by education. J Health Econ.

[30] Sapolsky RM (1995). Social subordinance as a marker of hypercortisolism. Some unexpected subtleties. Ann N Y Acad Sci.

[31] Steptoe A, Marmot M (2002). The role of psychobiological pathways in socio-economic inequalities in cardiovascular disease risk. Eur Heart J.

[32] Carlsen F, Kaarboe OM (2015). The relationship between educational attainment and waiting time among the elderly in Norway. Health Policy.

[33] Smith JP (1999). Healthy bodies and thick wallets: the dual relation between health and economic status. J Econ Perspect.

[34] Richardson J, Iezzi A, Khan MA (2015). Why do multi-attribute utility instruments produce different utilities: the relative importance of the descriptive systems, scale and ‘micro-utility’ effects. Qual Life Res.

[35] Gamst-Klaussen T, Chen G, Lamu AN, Olsen JA (2016). Health state utility instruments compared: inquiring into nonlinearity across EQ-5D-5L, SF-6D, HUI-3 and 15D. Qual Life Res.

[36] Khalatbari-Soltani S, Maccora J, Blyth FM, Joannès C, Kelly-Irving M (2022). Measuring education in the context of health inequalities. Int J Epidemiol.

[37] Garratt AM, Hansen TM, Augestad LA, Rand K, Stavem K (2022). Norwegian population norms for the EQ-5D-5L: results from a general population survey. Qual Life Res.

[38] van Hout B, Janssen MF, Feng YS, Kohlmann T, Busschbach J, Golicki D (2012). Interim scoring for the EQ-5D-5L: mapping the EQ-5D-5L to EQ-5D-3L value sets. Value Health.

[39] Shmueli A (2003). Socio-economic and demographic variation in health and in its measures: the issue of reporting heterogeneity. Soc Sci Med.

[40] Knott RJ, Lorgelly PK, Black N, Hollingsworth B (2017). Differential item functioning in quality of life measurement: an analysis using anchoring vignettes. Soc Sci Med.

