# Supplementary material for: Inequality in quality-adjusted life expectancy by educational attainment in Norway: an observational study
Source: BMC Public Health. 2023 May 3;23:805. doi: 10.1186/s12889-023-15663-2 (PMC10155341; doi:10.1186/s12889-023-15663-2)
Supplement: Supplementary file 1 — Additional file 1: Methods to calculate life tables and QALE. [file 12889_2023_15663_MOESM1_ESM.docx]

Supplementary material for ‘Inequality in quality-adjusted life expectancy by educational attainment in Norway: an observational study’

**Methods to calculate life tables and QALE**

LE at age 40 is calculated as

$${LE}_{40}=\frac{\sum_{x=40}^{106} \left( l_{x}-0.5 x d_{x} \right)}{l_{x}}$$

where

m_x_ (=deaths observed / population) is the age-specific death rate at age x,

$q_{x}(=m_{x}/(\left( 1+0.5 \right) x m_{x})$is the conditional probability that an individual entering the age interval x will die in this age interval,

$l_{x}(= l_{x-1}-d_{x-1})$ is the proportion of individuals alive at the start of interval x, and $l_{40}$ = 1,

$d_{x} (= q_{x} x l_{x})$ is the proportion of individuals who will die during interval x.

Following Sullivan, QALE at age 40 is calculated as

$$QA{LE}_{40}=\frac{\sum_{x=40}^{106} \left( l_{x}-0.5 x d_{x} \right) x h_{x}}{l_{x}}$$

where

$h_{x}$= mean HRQoL at age x.

To reflect uncertainty in $q_{x}$ and $h_{x}$ we conduct 1,000 simulations where in each simulation we a) resample with replacement the respondent-level data from the Tromso study to calculate $h_{x}^{*}$, and b) we perform a random draw from a beta distribution so that $q_{x}^{*}$ = beta(a,b), where

$$a = (((1-q_{x})/ var(q_{x}))-(1/q_{x})) * {q_{x}}^{2}$$

$$b = a * (1/q_{x}-1)$$

and $var\left( q_{x} \right)= \left( {q_{x}}^{2} * \left( 1-q_{x} \right) \right) x deaths$, and c) we recalculated LE and QALE based on $q_{x}^{*}$ and $h_{x}^{*}$.

All calculations were conducted separately by educational attainment and sex.
