# Supplementary material for: Inequality in quality-adjusted life expectancy by educational attainment in Norway: an observational study
Source: BMC Public Health. 2023 May 3;23:805. doi: 10.1186/s12889-023-15663-2 (PMC10155341; doi:10.1186/s12889-023-15663-2)
Supplement: Supplementary file 2 — Additional file 2: Table S1. Comparison of EQ-5D-5L utility scores in two surveys of the Norwegian general population calculated using different value sets. [file 12889_2023_15663_MOESM2_ESM.docx]

Supplementary material for ‘Inequality in quality-adjusted life expectancy by educational attainment in Norway: an observational study’

Table S1: Comparison of EQ-5D-5L utility scores in two surveys of the Norwegian general population calculated using different value sets

|  | Tromsø study - wave 7 | | |  | Garratt et al. 2022 | |
| --- | --- | --- | --- | --- | --- | --- |
|  | N | mn-WEPP (2022) algorithm | van Hout (2012) algorithm |  | N | van Hout (2012) algorithm |
| *Males* |  |  |  |  |  |  |
| 40-49 | 2943 | 0.909 | 0.850 |  | 258 | 0.786 |
| 50-59 | 2665 | 0.905 | 0.844 |  | 253 | 0.831 |
| 60-69 | 2387 | 0.910 | 0.850 |  | 214 | 0.826 |
| 70-79 | 1201 | 0.916 | 0.859 |  | 166 | 0.805 |
| 80+ | 277 | 0.876 | 0.812 |  | 106 | 0.746 |
|  |  |  |  |  |  |  |
| *Females* |  |  |  |  |  |  |
| 40-49 | 3251 | 0.886 | 0.823 |  | 272 | 0.790 |
| 50-59 | 3105 | 0.881 | 0.814 |  | 264 | 0.767 |
| 60-69 | 2542 | 0.894 | 0.828 |  | 215 | 0.806 |
| 70-79 | 1238 | 0.874 | 0.809 |  | 156 | 0.781 |
| 80+ | 331 | 0.819 | 0.757 |  | 68 | 0.697 |
